# A Case of Large, Hemodynamically Significant Abdominal Wall Hematoma Following Paracentesis in a Cirrhotic Patient

**DOI:** 10.7759/cureus.1483

**Published:** 2017-07-17

**Authors:** Samina Afreen, Usha Deonarine, Funmilola Ogundipe, Alicia Thomas

**Affiliations:** 1 Internal Medicine, Howard University Hospital; 2 Critical Care Medicine, Howard University Hospital

**Keywords:** paracentesis, cirrhosis, hematoma, renal failure, child pugh, coagulopathy, hemorrhagic

## Abstract

Paracentesis is a safe procedure with severe bleeding occurring in less than 1% of cases. Paracentesis is often times performed as an outpatient procedure. Hemorrhagic complications can be rapidly fatal if not diagnosed and treated in a timely fashion. We present the case of a 55-year-old female with decompensated cirrhosis who developed hemodynamically significant bleeding post paracentesis. This case brings up the question whether certain patients who undergo paracentesis should be admitted for close observation for at least 24 hours after the procedure. It also identifies the need for more research into pre-operative risk factors in cirrhotics that predisposes them to severe bleeding.

## Introduction

Large volume paracentesis has been considered a safe procedure, carrying approximately 1% risk of complications [1]. Complications that can occur include local infection, abdominal wall hematomas, intraperitoneal haemorrhage, and intestinal perforation [1]. Despite the increased coagulopathy in patients with hepatic dysfunction, severe bleeding is rare, occurring in less than 1% of cases [2]. The most common etiologies of major bleeding are abdominal wall hematoma and hemoperitoneum [3].

## Case presentation

**Figure 1:**
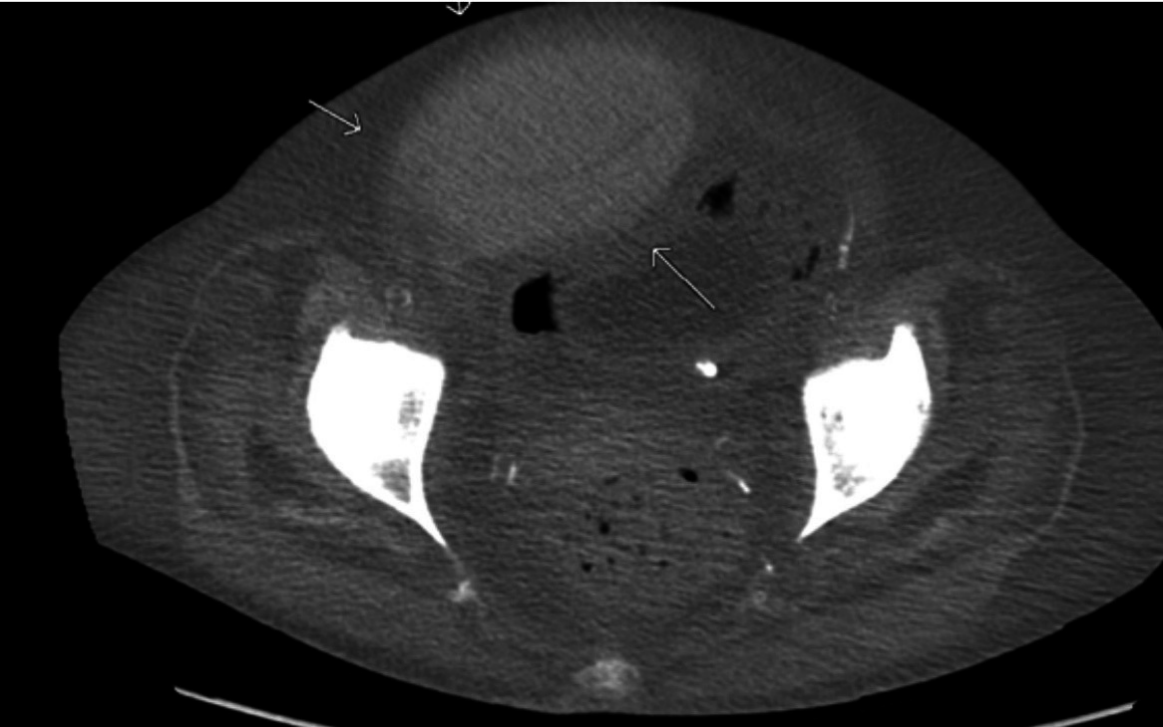
Computed tomography (CT) scan of abdomen and pelvis without contrast showing a 12.6 cm × 9.3 cm × 5.6 cm large mass along the anterior lower abdominal wall representing a bleed in the rectus muscle.

A 55-year-old African American female with past medical history of portal hypertensive gastropathy, cirrhosis secondary to hepatitis C, hypertension, portal hypertension, type 2 diabetes mellitus, presented with altered mental state of two days duration. Prior to the change in her mentation, the patient experienced three weeks of abdominal pain, black stool and vomiting. On examination, she was drowsy and ill-looking. She had pallor, tender distended abdomen, positive shifting dullness, generalized edema. Her blood pressure was 183/77 mm Hg, pulse rate was 109 beats/minute, temperature was 98.3°F, respiratory rate was 20 breaths/minute, and she was saturating 100% on 2 liters of oxygen. Laboratory data showed hemoglobin of 3.9 g/dl, white blood cell (WBC) count of 17.5 × 10^3^/microliter, platelet count of 374 × 10^3^/microliter, creatinine of 6.8 mg/dl, blood urea nitrogen (BUN) of 86 mg/dl, bicarbonate of 18 mEq/l, albumin of 1.4 g/dl, ammonia of 153 mcg/dl and international normalized ratio (INR) of 2.02. Child Pugh score was 13 and Model for End-Stage Liver Disease (MELD) score was 28 on admission. Computed tomography (CT) scan of the brain was normal. CT scan of the abdomen revealed anasarca with massive ascites and moderate left-sided pleural effusion. The patient was admitted for management of acute hepatic encephalopathy, upper gastrointestinal (GI) bleed, hypertensive emergency and decompensated cirrhosis. She was transfused with packed red blood cells for a goal hemoglobin of 7 g/dl and started on labetalol drip. Vancomycin, piperacillin-tazobactam, pantoprazole and lactulose were started. Endoscopy showed severe esophagitis with multiple eschar and a dominant ulcer crater at gastroesophageal junction with clot and evidence of recent bleeding. Epinephrine injection was administered at the site of bleeding and ulcer crater was cauterized. On day five, the patient was given one bag of fresh frozen plasma and left-sided thoracentesis was performed, which was uneventful. The patient underwent CT-guided large volume paracentesis on day six for persistent abdominal discomfort and tense ascites. One thousand eight hundred and thirty milliliters of clear ascitic fluid was removed. On the day of paracentesis, INR was 1.3, platelets were 142 × 10^3^/microliter, WBC count was 17.4 × 10^3^/microliter, hemoglobin was 10.1 g/dl, BUN was 21 mg/dl and creatinine was 1.5 mg/dl. After 10 hours of the procedure, the patient developed severe abdominal pain and hemoglobin was noted to have fallen to 5.4 g/dl. Repeat CT scan of the abdomen showed large mass measuring 12.6 cm × 9.3 cm × 5.6 cm along the anterior lower abdominal wall (Figure 1). This mass was dense with Hounsfield density of 60.4, representing hematoma in the rectus abdominal muscle.

The patient was transfused three units packed red blood cells and one bag of fresh frozen plasma. Gel foam was inserted in the tract where a needle was inserted for paracentesis, after which hemostasis with stabilization of hemoglobin was attained.

## Discussion

Ascites in liver cirrhosis is mainly medically managed by salt restriction and diuretics. However, randomized controlled trials have shown that large volume paracentesis (LVP) is safer and more effective than therapy with diuretics for the treatment of tense ascites [4]. Patients treated with LVP supported by plasma volume expansion have shortened hospitalization, better-preserved systemic hemodynamics and renal function. Additional benefits include improvement in hepatic hemodynamics, decreased risk of developing spontaneous bacterial peritonitis and less frequent hepatic encephalopathy [5]. Moreover, LVP is very effective in relieving dyspnea in patients with tense ascites.

For paracentesis, a needle is inserted in the left lower quadrant of abdomen, 4-5 cm medial to the anterior superior iliac spine. Right lower quadrant is not commonly used because of the risk of trauma to the liver if it is enlarged. Generally, under sterile conditions and if performed in appropriate manner, complications are rare. However, complications including leakage of ascitic fluid, local infection, intestinal perforation and hemorrhagic complications may occur. Three types of hemorrhagic complications have been noted to occur: abdominal wall hematomas, hemoperitoneum and pseudoaneurysm. Abdominal wall hematoma is the most frequent hemorrhagic complication [6].

In an observational study performed by Mercaldi and Lanes, it was demonstrated that the use of ultrasound guidance when compared with blind drainage, decreased the risk of paracentesis associated bleeding by as much as 68% [7]. In cirrhotics with massive ascites, the presence of collateral circulation of the abdominal wall places them at risk of severe bleeding. Our patient had CT-guided paracentesis and no abdominal wall vessels were visualized during the needle insertion, hence decreasing the likelihood of injury to blood vessels on the abdominal wall. 

INR is used as a screening test for coagulopathy before performing most procedures. However, an increased incidence of bleeding has not been observed when the INR was pathologically increased in cirrhotics [4,6]. This is because there is a balanced decrease in both pro- and anti-coagulant factors synthesized by the liver and increased INR only reflects the decrease in procoagulant factors [8]. Our patient’s INR was elevated on admission but not on the day of paracentesis.

Fibrinolysis is often increased in liver disease and parallels the degree of liver dysfunction. Hyperfibrinolysis promotes premature clot dissolution and interferes with clot formation due to the consumption of clotting factors. Hyperfibrinolysis overlaps with accelerated intravascular coagulation and fibrinolysis which can be evident as a distinct clinical entity with intractable bleeding following puncture wounds or dental extractions, or on occasion without any recognizable trauma [9]. Thromboelastography can be used in advanced liver disease patients as a pre-op evaluation.

Similarly in patients with thrombocytopenia paracentesis is not considered unsafe, with many successful uncomplicated procedures being carried out with platelet counts below 50,000 [4].

As these complications are rare, it is difficult to predict which patient will develop these complications. De Gottardi, et al., and Pache and Bilodeau noticed higher rates of complications in patients with higher Child-Pugh score [6, 10]. They also noticed a correlation between the risk of hemorrhage and renal dysfunction rather than coagulopathy and thrombocytopenia. Our patient had high Child Pugh Score, thus going with the findings of the studies by De Gottardi, et al., and Pache and Bilodeau. Although qualitative platelet dysfunction associated with renal failure can contribute to bleeding risk, thrombocytopenia in itself has not been shown to have any correlation with increased risk of hemorrhagic complications. So, studies need to be done to find out why deranged renal function increases risk of hemorrhagic complication and whether it would be** **reasonable to use Desmopressin before performing paracentesis in patients with cirrhosis and renal failure.

## Conclusions

Abdominal paracentesis is often times performed as an outpatient procedure. Hemorrhagic complications, though rare, if occur, can rapidly be fatal if not diagnosed and treated in a timely fashion. Risk factors for hemorrhagic complications of paracentesis in cirrhotics need to be determined. It also needs to be determined whether those at risk patients should be admitted for close observation for at least 24 hours after paracentesis to monitor them for the development of life threatening hemorrhagic complications. This case also brings up the question whether patients undergoing paracentesis need to undergo certain special tests like thromboelastography to better risk stratify them. We recommend more research into pre-operative risk factors in cirrhotics that predisposes them to severe bleeding.
